# InguinoScrotal Hernias: Comparison of Minimally Invasive Approaches and Open Surgical Repair (The SCAR Study)

**DOI:** 10.3389/jaws.2026.15830

**Published:** 2026-04-10

**Authors:** Riccardo Caruso, Emilio Vicente, Yolanda Quijano, Jorge Zarate Gomez, Belen Porrero Guerrero, Álvaro Valdés de Anca, Maria Teresa Alonso Garcia, Álvaro Morales Taboada, Pablo Pastor Riquelme, Sergio Salido Fernandez, Javier Serrano Gonzalez, Ana Pilar Morante Perea, Valentina Ferri

**Affiliations:** 1 General Surgery Department, Hospital Universitario HM Sanchinarro, HM Hospitales, Facultad HM de Ciencias de la Salud, Universidad Camilo José Cela, Instituto de Investigación Sanitaria HM, Madrid, Spain; 2 General Surgery Department, Hospital Universitario Quirónsalud Madrid, Madrid, Spain; 3 General Surgery Department, Hospital Ramón y Cajal de Madrid, Madrid, Spain; 4 General Surgery Department, Hospital Universitario de la Princesa, Madrid, Spain; 5 General Surgery Department, Hospital Universitario Santa Cristina, Madrid, Spain; 6 General Surgery Department, Hospital General Universitario Gregorio Marañón, Madrid, Spain; 7 General Surgery Department, Hospital Universitario Fundación Jiménez Díaz, Madrid, Spain; 8 General Surgery Department, Hospital Universitario La Paz, Madrid, Spain; 9 General Surgery Department, Hospital Universitario de Torrejon, Madrid, Spain; 10 General Surgery Department, Hospital Universitario Fundación de Alcorcón, Madrid, Spain

**Keywords:** comparative study, minimally invasive, open surgery, scrotal hernia, study design

## Abstract

**Introduction:**

Inguinal hernia repair is one of the most common surgical procedures performed worldwide. Minimally invasive approaches (laparoscopic and robotic) are increasingly recommended owing to their favorable postoperative outcomes. However, their use in inguinoscrotal hernias remains limited. This study aims to evaluate and compare clinical outcomes of minimally invasive and open approaches in inguinoscrotal hernia repair.

**Methods and analysis:**

This national multicenter prospective observational cohort study will include patients undergoing elective or urgent repair of European Hernia Society-classified inguinoscrotal hernias (S1–S3) using open, laparoscopic, or robotic techniques. Adults (>18 years) from various hospitals in Madrid will be recruited. Data on patient demographics, intraoperative variables, postoperative complications, postoperative pain (Visual Analog Scale score), quality of life, and hernia recurrence over a 2-year follow-up will be collected. A minimum of 100 patients (50 per group) is required for detecting statistically significant differences, assuming 5% loss to follow-up.

**Ethics and dissemination:**

The HM Hospitales Ethics Committee has approved the study protocol. Written informed consent will be obtained from all participants. Results will be submitted to peer-reviewed journals and presented at national surgical conferences.

## Introduction

Inguinal hernia repair is among the most frequently performed surgeries globally, accounting for more than 20 million procedures performed annually [1]. Although most of inguinal hernias are straightforward to repair, inguinoscrotal hernias represent a particularly complex subset, characterized by the descent of the hernia sac into the scrotum. These hernias are associated with larger defect sizes, a more difficult dissection, increased operative times, and a higher risk of complications, including orchitis, testicular atrophy, and wound-related issues [2, 3]. In high-income countries, their incidence is relatively low, accounting for approximately 5%–6% of all groin hernias [4]. However, the rate of inguinoscrotal hernias is substantially higher in low- and middle-income regions owing to delayed diagnosis and limited access to elective hernia repair [5]. Therefore, they pose a considerable burden for both patients and healthcare systems, particularly in terms of morbidity and quality of life (QoL).

The introduction of minimally invasive approaches, including laparoscopic transabdominal preperitoneal (TAPP) and totally extraperitoneal (TEP) repair, has significantly advanced inguinal hernia surgery. Several randomized controlled trials and meta-analyses have indicated that minimally invasive surgery is associated with reduced postoperative pain, fewer wound complications, earlier return to work, and better cosmetic outcomes than open anterior repair [6–8]. Robotic-assisted hernia repair has emerged more recently and appears to provide comparable safety and feasibility, with potential advantages in terms of dexterity, ergonomics, and visualization [9].

Despite these developments, evidence specifically addressing inguinoscrotal hernias remains scarce. Most clinical trials have excluded or underrepresented patients with large inguinoscrotal defects, leaving a knowledge gap regarding the optimal surgical approach for this subgroup [10]. The European Hernia Society (EHS) guidelines acknowledge the potential advantages of minimally invasive approaches but highlight that their use in complex hernias, including inguinoscrotal types, should be limited to surgeons with sufficient expertise and in centers with adequate resources [11]. Currently, only small retrospective series or single-center studies have evaluated laparoscopic or robotic repair in inguinoscrotal cases, frequently underscoring longer operative times and higher technical demands, but with promising results regarding recovery and complication rates [12, 13].

The SCAR study aims to compare short- and long-term clinical outcomes of open versus minimally invasive (laparoscopic and robotic) approaches for inguinoscrotal hernia repair. Particularly, this study will evaluate perioperative complications, postoperative pain, hospital stay, QoL, and long-term outcomes (e.g., recurrence and chronic pain).

## Main and Secondary Hypothesis

We hypothesize that minimally invasive approaches—both laparoscopic and robotic—will lead to better early postoperative outcomes compared with open repair in patients with inguinoscrotal hernias. Specifically, we expect lower rates of surgical site infections and overall complications (as graded by the Clavien–Dindo classification), reduced postoperative pain as measured by the Visual Analogue Scale, and shorter hospital stays.

We also hypothesize that these advantages will translate into an improved early health-related quality of life, as assessed through the EQ-5D-5L questionnaire.

Regarding long-term results, we hypothesize that minimally invasive repair may be associated with lower rates of chronic pain and better long-term quality of life compared with open surgery.

Additionally, we expect that recurrence rates at 2 years will not significantly differ between minimally invasive and open approaches, reflecting comparable long-term durability of the repair.

## Methods and Design

The SCAR study is a prospective multicenter non-randomized observational cohort study conducted across eight centers in Madrid (Spain) specializing in abdominal wall surgery. The objective is to compare clinical outcomes of inguinoscrotal hernia repairs performed via minimally invasive approaches (laparoscopic or robotic) versus open surgery.

Local approval was necessary for the individual participating centers. Patients eligible to participate in the study will be approached by the investigators and subsequently recorded. All included patients will sign a written informed consent form before study initiation.

### Study Population and Eligibility Criteria

All consecutive patients undergoing surgical repair for inguinoscrotal hernia will be considered eligible when they meet all of the following criteria at the time of enrolment:

### Inclusion Criteria

• Age ≥18 years;
• Diagnosis of inguinoscrotal hernia classified as either S1, S2, or S3 according to the EHS guidelines [11];
• Indication for surgical repair performed either electively or in an urgent setting; and
• Surgical approach using either open, laparoscopic, or robotic technique according to the standard practice of each center.

### Exclusion Criteria

• American Society of Anesthesiologists (ASA) physical status >3;
• Inability to sign the informed consent and age <18 years; and
• Refusal to participate or provide signed informed consent.

### Sample Size Calculation and Justification

The sample size was calculated to detect clinically significant differences in postoperative outcomes between minimally invasive (laparoscopic or robotic) and open approaches for inguinoscrotal hernias. Specifically, the calculation was based on the hypothesis that minimally invasive approaches would decrease postoperative complication rates by 20% compared with open surgery and lead to a 2-point reduction in postoperative pain (measured using the Visual Analog Scale).

Assuming a 5% significance level (two-sided α) and 80% power (1−β), and accounting for an estimated 5% loss to follow-up, a total of 150 patients (50 per surgical group: open, laparoscopic, robotic) will be required. This sample will enable the detection of statistically and clinically meaningful differences across the three groups using analysis of vairance (ANOVA) or Chi-square tests as appropriate.

### Trial-Specific Interventions

This is a pragmatic observational study with no imposed modifications to the standard surgical techniques employed at participating institutions. Each center will perform inguinoscrotal hernia repair on the basis of their routine clinical protocols and surgeon expertise.

Open group: may encompass anterior (Lichtenstein) or preperitoneal approaches, with mesh use and fixation technique determined by the operating surgeon.

Laparoscopic and robotic group: comprises TAPP or TEP techniques.

No restrictions regarding mesh type or fixation method are imposed; however, all intraoperative details (mesh type, fixation, use of drains, surgical time, and conversion) will be recorded for future analysis. Surgeons are anticipated to have completed their learning curve in the selected approach. Postoperative management, including analgesia, discharge criteria, and follow-up, will follow each center’s standard practice. This pragmatic approach ensures the study reflects real-world clinical scenarios and facilitates generalizability of the findings. To identify any variability between centers and detect potential confounders, all perioperative data will be systematically collected.

### Outcome Measures

- Primary outcomes:
- The primary outcomes of this study encompass the postoperative outcomes.
  - Surgical site infection and surgical site occurrence will be analyzed, and general complication will be assessed and graded using the Clavien–Dindo classification system.
- Surgical site occurrence (SSO) was defined as any wound-related complication within 30 days after surgery, including both infectious and non-infectious events. SSO included seroma, hematoma, wound dehiscence, and SSI.
- Surgical site infections (SSI) were assessed within 30 days after surgery. SSI were classified as superficial, deep, or organ/space infections. Superficial SSI involved the skin and subcutaneous tissue, while deep SSI involved the fascia or muscle layers. Organ/space SSI referred to infections involving anatomical structures manipulated during surgery other than the incision.
  - Postoperative pain will be evaluated using the VAS.
  - The length of hospital stay will be recorded for all patients as a key marker of recovery.
  - Furthermore, the assessment of health-related QoL using the QoL questionnaires EuroQol EQ-5D-5LTM will continue.
  - Return to normal daily activities
- Secondary outcomes:
- The secondary endpoints will focus on long-term outcomes.
  - Evaluation of hernia recurrence
  - Chronic pain and health-related QoL will be assessed at long-term follow-up intervals.

### Data Collection and Analysis

#### Patient Baseline Characteristics

- Age
- Sex
- Body mass index (BMI)
- ASA physical status
- Comorbidities (as assessed using the Charlson Comorbidity Index)—will be recorded.
- Type of hernia and classification

#### Intraoperative Data

- Surgical approach (open, laparoscopic, or robotic)
- Surgical technique
- Operative time
- Mesh type
- Mesh size
- Space of mesh placement
- Conversion
- Hospital stay

#### Postoperative Data

- Surgical site events
- Postoperative adverse events (all occurrences other than surgical site occurrences, including ileus, pulmonary embolism, or pneumonia) according to the Clavien–Dindo Classification up to 30 days
- Duration of analgesic medication use
- Hospital readmission rate
- Sick leave duration, defined as the number of days until return to work or to normal daily activities
- Recurrence rate, both clinically and radiologically (via ultrasonography or computed tomography), will be evaluated at 6 months, 1 year, and 2 years.
- QoL using the EuroQol EQ-5D-5LTM Spanish version: patients will receive questionnaires at inclusion and at 1, 6, 12, and 24 months postoperatively by mail, digitally, or in person according to their preference. Using the EQ-5DTM questionnaire, all participants will be asked to score the following five aspects of health status: mobility, self-care, usual activities, pain/complaints, and mood (anxiety/depression). This questionnaire is considered the most representative QoL tool for cost-effectiveness–related studies [14].
- Pain intensity using the VAS
- Return to normal daily activities

#### Patient Timeline and Trial Visits

All patients scheduled for elective inguinoscrotal hernia repair (open, laparoscopic, or robotic approach) in the participating centers will be screened for eligibility and considered for study inclusion. Reasons for exclusion (whether owing to ineligibility or patient refusal) will be documented.

Patients who meet the inclusion criteria and can understand the scope of this study will be invited to participate. Written informed consent will be obtained after providing detailed information about the study objectives and procedures.

Baseline data collection will transpire during Visit 1, including demographic characteristics, comorbidities, clinical data, and the first QoL questionnaire. Visit 2 refers to the surgery day, where intraoperative and surgical details will be recorded. Postoperative outcomes and complications will be monitored during Visit 3 (discharge) and 4 (after 7 days). Moreover, any postoperative complication-related diagnostic or therapeutic interventions will be reported, and VAS scores will be calculated. To assess the persistence or recurrence of symptoms, long-term complications, reoperations, and QoL, follow-up will continue at Visit 5 (1 month), Visit 6 (6 months), Visit 7 (12 months), and Visit 8 (24 months) (Table 1).

**TABLE 1:** Trial visits and documented parameters.

Assessment	Trial visits						
	Baseline	Day 0	Day 1	Day 7	Day 30	6 months	1 and 2 years
	Visit 1	Visit 2	Visit 3	Visit 4	Visit 5	Visit 6	Visit 7 and 8
Eligibility criteria	X	​	​	​	​	​	​
Informed consent	X	​	​	​	​	​	​
Demographic and baseline characteristics	X	​	​	​	​	​	​
Surgical details	​	X	​	​	​	​	​
Taken pain medication	​	​	X	​	X	​	​
Postoperative complication	​	​	X	​	X	​	​
Readmission rate	​	​	X	​	X	​	​
Sick leave	​	​	​	​	X	X	​
QoL assessment	X	​	X	​	X	X	X
VAS	X	​	X	X	X	X	X
Recurrence	​	​	​	​	​	X	X

Abbreviations: POD, postoperative day; QoL, quality of life; CCI, comprehensive complication index; V, visit; VAS, visual analog scale.

### Data Management

All study variables will be documented in a secure electronic case report form (eCRF), developed specifically for this protocol and accessible through a dedicated online platform. Data from each participating center will be directly entered by local investigators or study coordinators and automatically transferred to a centralized database.

Each authorized researcher and study monitors will have individual digital access to the eCRF, enabling them to include new patients, update data, and review follow-up entries. All changes or additions in the eCRF system will be automatically logged in an audit trail.

To prevent data loss, monthly backup copies of the database will be generated. The eCRF system and the database will be securely stored and retained for at least 5 years following the study’s completion, with full compliance with GDPR and confidentiality standards equivalent to those used for medical records.

### Statistical Analyses

Statistical analysis will follow the intention-to-treat principle. Descriptive statistics will be employed for summarizing baseline characteristics and perioperative outcomes across the three surgical groups: open, laparoscopic, and robotic.

Univariate analyses (Chi-square or Fisher’s exact test and ANOVA or Kruskal–Wallis test for categorical and continuous variables, respectively) will be performed for comparing outcomes, including complication rates, recurrence, surgical site infections, and hospital length of stay.

To control for potential confounders, multivariate logistic regression models will be applied, particularly for primary outcomes, including postoperative complications (classified using the Clavien–Dindo classification system and Comprehensive Complication Index), hernia recurrence, and chronic postoperative pain.

Time-to-event outcomes (e.g., time to recurrence) will be analyzed using Kaplan–Meier survival curves and compared using log-rank tests. To assess predictors of recurrence or reoperations, Cox proportional hazards models will be employed.

Subgroup analyses will be considered on the basis of patient age, BMI, hernia type (primary vs. recurrent), and surgical approach. P < 0.05 will be considered statistically significant.

To assess data quality, protocol adherence, and recruitment balance across centers, an interim descriptive analysis may be performed following the inclusion of 50% of the expected patient sample.

### Duration and Schedule

#### Phase 1: Patient Inclusion

The recruitment period for patient inclusion in the clinical trial will span 22 months. As the variable “recurrence” requires a maximum follow-up of 2 years postoperatively, this study will conclude at 24 months following the inclusion of the last patient. Therefore, the total study duration, from the inclusion of the first patient to the collection of data from the last patient, will be 46 months.

#### Phase 2: Outcome Assessment

Integrated evaluation of results, encompassing auditing of the selected clinical cases and publication of the findings.

### Publications

#### Two Publications Are Planned for This Study

Short-term outcome publication, which will be reported following the recruitment of the last patient.

Two-year outcome publication, which will be reported at the end of the follow-up period, 24 months postoperatively.

## Discussion

Considering the complexity of inguinoscrotal hernias and the paucity of robust evidence, prospective studies evaluating outcomes of different surgical approaches in this specific subgroup are needed. Open repair remains broadly employed and is considered technically straightforward in experienced hands; however, it may be associated with higher wound morbidity rates and postoperative pain in large hernias [14]. Minimally invasive approaches, although technically demanding, may decrease postoperative morbidity and enhance long-term QoL when adapted to the anatomical challenges of the inguinoscrotal subtype.

To provide high-quality real-world data on the perioperative and long-term outcomes of open, laparoscopic, and robotic repairs, the Study on Comparison of open versus minimally invasive Approaches in inguinoscrotal heRnia repair (SCAR) trial has been designed as a multicenter prospective observational cohort study. By systematically collecting data across several high-volume abdominal wall surgery centers, the study aims to fill the existing knowledge gap and guide future guideline recommendations for this uncommon but clinically significant hernia subgroup.

## Data Availability

The raw data supporting the conclusions of this article will be made available by the authors, without undue reservation.
